# Three-gradient regular solution model for simple liquids wetting complex surface topologies

**DOI:** 10.3762/bjnano.7.129

**Published:** 2016-10-04

**Authors:** Sabine Akerboom, Marleen Kamperman, Frans A M Leermakers

**Affiliations:** 1 Physical Chemistry and Soft Matter, Wageningen University, Stippeneng 4, 6708 WE Wageningen, Netherlands

**Keywords:** inverse opal, regular solution model, self-consistent field theory, surface topology, wetting

## Abstract

We use regular solution theory and implement a three-gradient model for a liquid/vapour system in contact with a complex surface topology to study the shape of a liquid drop in advancing and receding wetting scenarios. More specifically, we study droplets on an inverse opal: spherical cavities in a hexagonal pattern. In line with experimental data, we find that the surface may switch from hydrophilic (contact angle on a smooth surface θ_Y_ < 90°) to hydrophobic (effective advancing contact angle θ > 90°). Both the Wenzel wetting state, that is cavities under the liquid are filled, as well as the Cassie–Baxter wetting state, that is air entrapment in the cavities under the liquid, were observed using our approach, without a discontinuity in the water front shape or in the water advancing contact angle θ*.* Therefore, air entrapment cannot be the main reason why the contact angle θ for an advancing water front varies. Rather, the contact line is pinned and curved due to the surface structures, inducing curvature perpendicular to the plane in which the contact angle θ is observed, and the contact line does not move in a continuous way, but via depinning transitions. The pinning is not limited to kinks in the surface with angles θ_kink_ smaller than the angle θ_Y_. Even for θ_kink_ > θ_Y_, contact line pinning is found. Therefore, the full 3D-structure of the inverse opal, rather than a simple parameter such as the wetting state or θ_kink_, determines the final observed contact angle.

## Introduction

**Figure 1:**
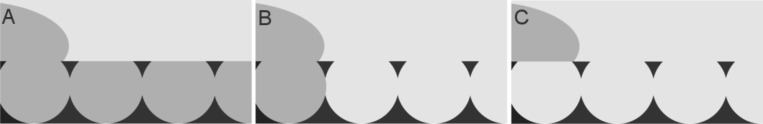
Schematic representation of the three wetting states on an inverse opal, A) impregnated state, B) Wenzel state, C) Cassie–Baxter state.

Wetting of surfaces is a key feature for many applications. The wetting properties of a surface depend on both the material and the surface topography. A famous example is the surface of a lotus leaf: Although the material of the leaf is hydrophilic (contact angle on a smooth substrate θ_Y_ < 90°), the structured surface is hydrophobic (apparent contact angle θ > 90°) [1]. Recently, different surface structures have been designed and fabricated from hydrophilic materials that show hydrophobic contact angles [2–10]. An example is an inverse opal as schematically shown in Figure 1. Our group recently reported an increase of θ from ca. 80° to ca. 110° for an inverse opal of polypyrrole [10].

Our study is targeted to obtain (close to) molecular level insight in the wetting features of such surfaces using a simplistic modelling toolbox based on regular solution theory. To understand the increase in θ, which is observed at macroscopic length scales, details about the microscopic scale should be considered. For the simplest case in which a water droplet wets the structured surface on a microscopic level with its preferred angle θ_Y_ (see Figure 1B), the apparent contact angle, θ_W_ is given by [11]

[1]
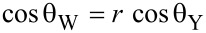


with *r* the roughness of the surface (true contact area/projected area). This is called the Wenzel state, and it always magnifies the underlying wetting properties: θ decreases for hydrophilic materials and increases for hydrophobic materials. As the structured surfaces of interest, which are composed of a hydrophilic material, show an increase in θ, this implies that the droplet in these systems cannot be in the Wenzel state, or that the assumption of this model, namely that the parameter *r* captures all features of a surface topography relevant for the final droplet shape, is too simplistic.

A possible explanation of the increase in θ on structured surfaces, is air entrapment [12–13]. Air acts as hydrophobic patch (θ_Y_ for the water/air interface is 180°), and these patches lower the average surface energy of the surface (see Figure 1C) [14–15]. The resulting apparent contact angle for this so-called Cassie–Baxter state is then given by [16]

[2]
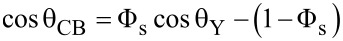


with Φ_s_ the fraction under the droplet that is in contact with the solid and (1 − Φ_s_) the fraction under the droplet in contact with air. This approach thus defines the solid as a new material with a different effective surface energy on a macroscopic scale, and does not entail details about the droplet shape close to the surface structures on a microscopic level.

**Figure 2:**
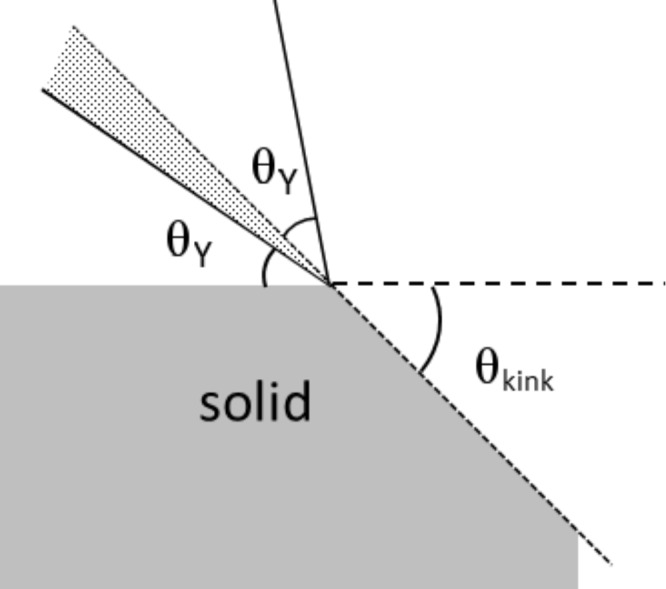
Surface structure induced contact line pinning in 1D: pinning occurs when θ_Y_ < θ_kink_ (dotted area). The two θ_Y_ indicate the contact angle on the surface before and after the droplet has reached the kink.

Another explanation of the difference in θ for a structured and unstructured surface of the same material is contact line pinning [17–20]. The three-phase contact line is hereby immobilized. Apart from chemical heterogeneities (which will not be discussed here), pinning occurs for a simple 1D system when the contact line encounters a kink in the surface, indicated with angle θ_kink_ in Figure 2. If θ_Y_ < θ_kink_, the angle of the droplet with respect to the surface should exceed θ_Y_ in order to wet the surface after the kink (dotted area in Figure 2), and the droplet is thus pinned.

However, pinning cannot result in any arbitrary shape. The mean curvature *J* of the liquid/vapour (L/V) interface of a droplet, is related to the pressure difference across the L/V interface, Δ*P,* and the interfacial tension γ according to the Young–Laplace equation [17]:

[3]
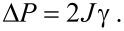


Δ*P* and γ can be considered constant for a droplet (neglecting small curvature corrections, the deformation due to gravity and the near surface contributions expressed in the disjoining pressure), thus *J* should also be constant [13]. This implies that if the surface structure induces a curvature in one direction due to pinning, this should be compensated by the opposite curvature in the perpendicular direction. Hence, if structuring of a surface induces a noticeable curvature of the droplet parallel to the surface, this should lead to deformations of the droplet perpendicular to the surface. As the latter curvature is coupled to the apparent contact angle, which is commonly measured perpendicular to the surface, we notice contact angle variations.

The feature of hydrophilic surfaces showing hydrophobic contact angles is, for reasons mentioned above, often linked to re-entrant angles of the surface structures [21–22]. An additional argument besides pinning, is that the liquid/air interfacial area should increase upon penetration of the liquid, creating more liquid/vapour interface [13]. This may imply that air entrapment occurs, even for hydrophilic materials [12,23].

Since it is difficult to observe local curvatures in the three phase contact line experimentally [18,24–25], in this study we turn to ‘experiments in silico’. The present study is targeted to obtain insight in the wetting features of surfaces of hydrophilic materials that show hydrophobic contact angles and to differentiate between air entrapment and contact line pinning using a modelling approach.

Macroscopic approaches such as solving the Young–Laplace equation [26–27], minimizing the availability [28], or using geometry and energy [12] to find the droplet shape, do not take molecular details into account, and often require the contact angle as input parameter. Furthermore, air entrapment and coalescence [29] cannot be obtained by solving the Young–Laplace equation, and surfaces with re-entrant curvatures give impossible solutions [29]. Phase field methods [29], molecular dynamics (MD) [23,30–32], and mesoscopic lattice-Boltzmann (LB) models [33–37] are a viable option for this problem, but are challenging because wetting on complex structures involves multiple length scales [38] and the time needed to converge to a solution can be long [31,38].

In this paper we focus on the very well known regular solution theory, which is frequently used throughout the field of physical chemistry, but not so often applied for studying wetting on complex surface topologies in three dimensions. Our models can be solved using a surprisingly simple algorithm (e.g., a Pikar iteration) on a desktop PC in a few minutes CPU time. However, similar to some of the theoretical approaches mentioned above, there are limitations with respect to the size of the systems that realistically can be considered. Albeit these limitations can easily be lifted by a factor of ten when the equations are solved using modern supercomputer facilities (which we here did not do). Here we focus on equilibrium and metastable states, which allows us to consider both advancing as well as receding contact angles. Even though the regular solution model is very well known, we will start by giving some backgrounds and highlights of the regular solution model. This gives us an opportunity to fix some of our parameters in the system. We then present the model and study the wetting of inverse opal structures.

## Results and Discussion

### Regular solution theory

The start is a lattice model wherein the sites with linear length *b* are arranged in a cubic lattice geometry, that is, each cell has *Z* = 6 neighbours. Let there be *M* sites in the system and thus the volume is given by *V* = *Mb*^3^. Sites are either filled by a solvent molecule, or the site is empty. The latter sites are said to be vacant and the number of vacant sites is *N*_V_. The remaining sites are filled by solvent and hence there are *N* = *M* − *N*_V_ sites filled. It is assumed that the solvent molecules only interact with each other when they occupy neighbouring sites and in this setting it is common to introduce the dimensionless Flory–Huggins interaction parameter, which is an Archimedean-like parameter needed for unlike contacts:

[4]
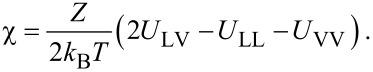


A positive value means that LL contacts and VV ‘contacts’ are favoured over LV ones and this implies a tendency towards demixing. When we assume random mixing (mean-field approximation) we can evaluate the mixing interaction energy in the system by *U*_mix_ = *N*χφ_V_, where we ignored boundary effects and φ_V_ = *N*_V_/*M* is the volume fraction of vacancies. The entropy of mixing can be evaluated when we assume once more that the sites are randomly filled by solvent. The total number of ways to arrange the fluid and the vacancies is given by 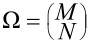 and the mixing entropy is found by *S*_mix_ = −*k*_B_·lnΩ = −*k*_B_(*N*·ln φ + *N*_V_·ln φ_V_) with φ = *N*/*M* and *k*_B_ the Boltzmann constant. The free energy of mixing is given by *F*_mix_ = *U*_mix_ – *T*·*S*_mix_. Introducing the dimensionless free energy density *f* = *F*_mix_/(*Mb*^3^*k*_B_*T*) wherein the thermal energy *k*_B_*T* and the volume *V* are used to reduce the free energy, we obtain the well-known regular solution free energy density:

[5]
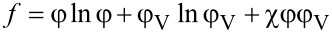


with φ + φ_V_ = 1. The first two terms are negative and promote the mixing of the solvent and vapour. The last term drives the demixing. The critical conditions are found by setting the second and third derivatives of the free energy density (Equation 5) with respect to the volume fraction of liquid to zero. From such analysis it is found that there is a solubility gap as soon as χ > χ^cr^ = 2. By symmetry the critical density φ^cr^ = 1/2.

### Liquid/vapour interface

Very famous is the extension of the regular solution theory to the description of the L/V interface. In the footsteps of van der Waals [39] we like to find the density profile across a L/V interface φ(*z*). Here *z* is a (lattice) coordinate running perpendicular to the interface. We set *z* = 0 at the interface and consider a lattice model with layer numbers *z* = −*M*, −(*M* − 1),…,−1, 0, 1,…, *M* − 1, *M*. The boundary layers −*M* and *M* are taken large enough so that the interface is not perturbed. We generalise Equation 5 and define a dimensionless free energy *F* as follows

[6]
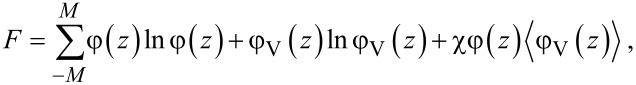


where it is understood that the mean-field approximation is now applied along lattice layers. The angular brackets in the last term indicate that in the interaction term “curvature” information is included, which is needed to evaluate the number of liquid–vacancy contacts in the presence of density gradients. In continuous language, we need to introduce

[7]
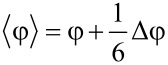


in the interaction term, which on a lattice and in a one-gradient functional of Equation 6, translates to a local averaging operation:

[8]
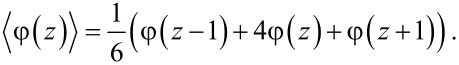


**Figure 3:**
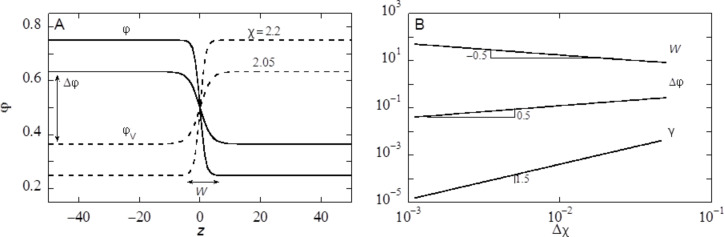
A) Examples of volume fraction profiles across a liquid/vapour interface found numerically by exact minimisation of Equation 6, with the interaction parameter χ = 2.05 and χ = 2.2 as indicated. B) Width of the interface (W), density difference between the two phases (Δφ) and dimensionless surface tension (γ) as function of Δχ = χ − 2 in double logarithmic coordinates.

The target is to find the best volume fraction profiles that optimise the free energy *F*. Results are summarised in Figure 3.

Two volume fraction profiles are presented in Figure 3A, which were found numerically by minimizing Equation 6, for two values of χ, not far from but above χ^cr^ = 2. We have set the liquid phase at negative values of *z*, whereas the vapour is at positive *z*. The position of the interface is set at *z* = 0 found by searching for φ = 0.5 (in three-gradient results we will find the interface by the same criterion). The profiles follow very accurately the tanh dependence (see Figure 3A for numerical results). We note that for χ = 2.2 used below, these results deviate from the analytical predictions. Far from the interface the volume fraction profile levels off to the binodal values. The difference in volume fractions between the binodal values, here defined by Δφ = φ(−*M*) − φ(*M*), is indicated in Figure 3A. The width *W* of the interface is numerically found by intersection of the tangent line at *z* = 0 with the binodal value. We can evaluate the surface tension γ, which is given in units *k*_B_*T*/*b*^2^*,* numerically, as discussed in section S1 of Supporting Information File 1.

In Figure 3B we prove that near the critical point (i) the surface tension, (ii) the width of the interface and (iii) Δφ as found by our numerical solution accurately obey scaling relations with respect to the difference to the critical point Δχ = χ − 2*.*

Interestingly, near the critical point there is an analytical route to optimise the free energy *F* [40]. In short, near the critical point the density of the liquid (and thus also for the vacancies) is never far from the critical value. Introducing an order parameter φ = φ – 0.5, we can write *F* as a function of the order parameter and then Taylor series expand the logarithms up to the fourth order in the order parameter. As a result we obtain a Landau free energy in terms of the order parameter. An Euler–Lagrange optimisation then leads to the famous tanh-profile already known by van der Waals. We do not go into these details and mention that fully in line with the numerical results presented in Figure 3B the scaling exponents as found by this analytical route are in line with the numerical results: For the surface tension the (mean field) value is −3/2, it is 1/2 for the width of the interface, while the difference in densities of the two phases vanishes with an exponent −1/2 [40].

Our aim is to present results that are relevant for the water/vapour system. Of course a symmetric lattice model falls short in this respect, because it assumes that as much water will be in the vapour phase as free volume will be in the water phase. The symmetry can only be broken in a more elaborate model wherein water is more realistically represented. We mention that such an approach is (at least in principle) possible, but here we choose not to go into such complications. We know that at ambient temperatures the water/vapour system is not near critical. Indeed it is very far from critical. Hence it is necessary to choose a sufficiently high χ value. In a lattice model it is advised to keep the width of the interface larger than the size of a lattice site (i.e., *W* > *b*). In the other limit one experiences many so-called lattice artefacts, which may frustrate the analysis of the shape of a droplet on top of a structurally complex surface. For this reason we choose here χ = 2.2 for the liquid/vapour interactions, unless stated otherwise. For this value the width of the interface *W* is approximately 8 in units *b*. As the width of an air-with-water interface is just a few angstroms [41], we may infer that when the water/vapour system is the target of our calculations, the corresponding value of *b* is a value less than an angstrom. Again we accept deviations from the air/water system and advice to consider the value of *b* to be in the order of a few angstroms (say 0.2 nm). The fraction of liquid in the liquid-rich phase for χ = 2.2 is about 0.7515, and the fraction of liquid in the vapour-rich bulk phase has the binodal value φ^#^ = 0.2485. Again these values differ dramatically from our experimental system of water in air at 100% relative humidity. Finally, the interfacial tension in this system is given by γ = 0.03326 in units *k*_B_*T*/*b*^2^, which translates with *b* = 0.2 nm to 3.422 mN/m. This value is smaller than the known value for water. All these differences with respect to our experimental system are accepted as we search only for scenarios. For ease of reference we may call the liquid-rich phase “water” and the vacancy-rich phase “vapour”.

### Droplet on an unstructured solid

Still using the one-gradient approach, it is possible to study wetting phenomena using the regular solution model. We need remarkably few modifications in the system. The only issue is that we need to introduce a substrate. To do so, we first specify the lattice coordinates *z* = 0, 1, 2,…, *M*, and introduce a surface component S as a boundary condition, that is, we choose φ_S_(0) = 1 and φ_S_(*z*) = 0 for all *z* > 0. The liquid and the vapour are allowed to be in the half-space *z* > 0. We have in principle two new interaction parameters χ_LS_ and χ_VS_ by introducing a “third” component.

Without losing generality we can set χ_VS_ = 0, and keep χ_S_ = χ_LS_ to specify the preferential adsorption of the liquid component on the surface. A negative value means that the solvent has a preference to sit next to the surface over the vapour. At χ_S_ = 0 we expect a contact angle of 90°. Hydrophobic surfaces are modelled when χ_S_ > 0. We will mostly restrict ourselves to hydrophilic surfaces, thus to χ_S_ < 0.

In the case of a L/V system next to a surface the regular solution free energy assumes the form

[9]
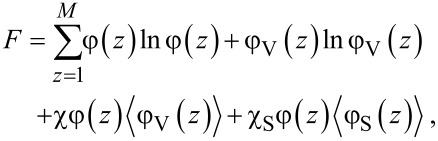


where it is understood that the last term is only non-zero when *z* = 1, where it assumes the value 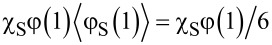.

There are several routes to study wetting. Our preference goes to study so-called adsorption isotherms. Of course we need a solubility gap and thus χ > 2 (we use a value of 2.2 throughout). Next, we consider a specific value of χ_S_ < 0 and specify a given amount of solvent 
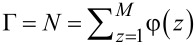
 in the system. We solve the self-consistent field equations and obtain the optimised density profile φ(*z*). Far from the surface, the density profile converges to the bulk value φ^b^. The adsorbed amount (surface excess) of liquid is found by:

[10]
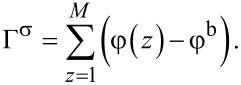


We focus on how the adsorption isotherms, Γ^σ^(φ^b^), behave near the bulk binodal φ^#^. When upon the approach of the bulk binodal the adsorbed amount simply increases and diverges at the binodal value, we have a complete wetting situation and the contact angle is zero. Alternatively, the isotherm crosses the binodal at a finite value of the adsorbed amount, that is, for an amount Γ^#^ = Γ^σ^(φ^b^) < ∞. We refer to this first crossing as the “microscopically thin film” adsorbed at the surface S. By means of a van der Waals loop the isotherm then returns to the binodal and approaches the infinite adsorbed amount upon the final approach towards the bulk binodal. We refer to this adsorbed amount as the “macroscopically thick film” on the surface. Such situation is typical for partial wetting states, where the macroscopically thick film represents the situation under a drop, and the thin film is found far away from the drop where a gas-like film resides on the substrate. As for each solution along the isotherm we have the surface tension accurately available from the self-consistent field (SCF) solution, we can find the contact angle from Young’s law:

[11]
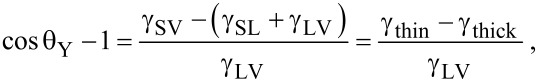


where all interfacial tensions are computed for systems in which the chemical potential is that corresponding to the bulk binodal. The value of γ_thin_ is found from the first crossing of the binodal, and γ_thick_ is the surface free energy in the system when there is a very thick adsorbed layer at the surface. Hence, we can obtain contact angle information without explicitly generating droplets. In passing we mention that for not too small droplets the contact angle as obtained by a three-gradient analysis (as used below) gives identical contact angles as the ones that follow from Equation 11, which used information from one-gradient regular solution models.

**Figure 4:**
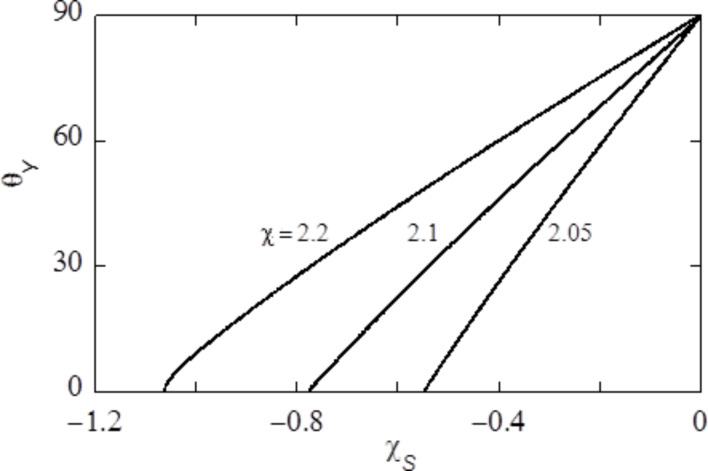
Contact angle of liquid on flat solid, θ_Y_ as function of the interaction parameter of the liquid with the solid, γ_S_, for three different values of the interaction parameter of the liquid with the vapour, γ.

The contact angle of a liquid droplet θ_Y_ is calculated for various adsorption strengths χ_S_ and for different strengths of interaction between liquid and vapour χ (Figure 4). The more negative χ_S_, the more favourable the interaction between S and L, and the more the droplet spreads, resulting in smaller θ_Y_. Eventually, the liquid prefers to wet the solid completely, i.e., θ_Y_ = 0°. At χ near the critical value of 2, the droplet enters the complete wetting regime (θ_Y_ = 0°) already for very low values of the surface affinity χ_S_. For strong segregations the interfacial energies increase and we need larger adsorption energies to enforce wetting.

In this paper we aim to mimic a polypyrrole surface for which the water contact angle of a smooth surface is about θ_Y_ = 80°. As we already selected χ = 2.2, we will be in the correct contact angle regime when we set the adsorption energies around χ_S_ = −0.2. Below we will always mention the strength of adsorption.

### Liquid condensation in parallel slit

The surface structure of an inverse opal consists of close to spherical cavities. In such structures we should anticipate the occurrence of capillary condensation or, alternatively, capillary drying. For this reason we use the regular solution model to study classical capillary condensation. To this end we consider a system that contains two surfaces. One at *z* = 0 and another one at *z* = *D* + 1. Hence φ_S_(*z*) = 0 for *z* = 1, 2,…, *D* and unity elsewhere. Now our regular solution free energy is given by

[12]
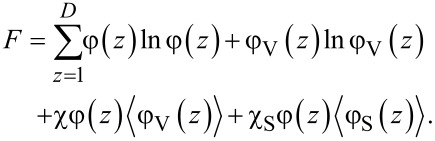


In this case the last term automatically accounts for the interactions with the surfaces, as it is non-zero for *z* = 1 and *z* = *D*. More specifically, *F* has two surface contributions χ_S_φ(1)/6 + χ_S_φ(*D*)/6.

**Figure 5:**
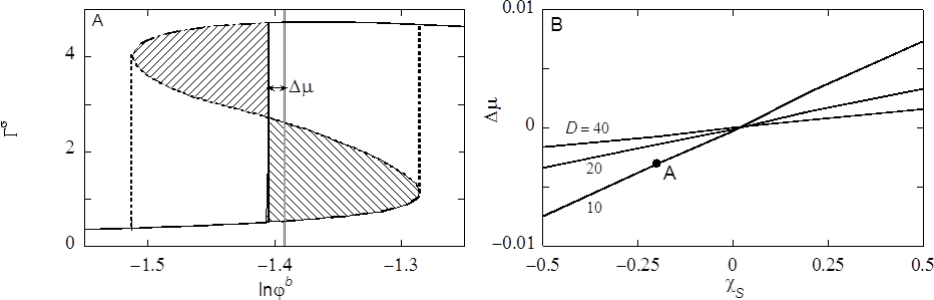
A) Adsorption amount Γ^σ^ of the liquid component as a function of the volume fraction of liquid in the vapour φ^b^ in lin–ln coordinates (*x*-axis is chemical potential), in a slit with surfaces that are a distance *D* = 10 apart and have an adsorption energy χ_S_ = −0.2. The metastable branches are dashed. The unstable part is dotted. The chemical potential µ^#^ is indicated as the grey vertical line. The solid vertical line is placed at the chemical potential where a step in the isotherm takes place (local binodal). This step is found by the Maxwell construction, that is by the equal area argument (the two shaded regions have the same area). The difference in chemical potential Δµ between the place of the step and the binodal is indicated. The vertical dotted lines are placed at chemical potentials corresponding to the spinodal points. B) The chemical potential where the step in the isotherm (compared to value of the bulk binodal) Δµ takes place (at equilibrium) as function of χ_S_ for slit distances *D* =10, 20 and 40 as indicated. The solvent–vacancy interaction is set to χ = 2.2. The label A on the curve for *D* = 10 corresponds to the condition used in panel A.

As there are two interfaces, we anticipate the adsorption of the liquid onto both surfaces simultaneously. We want to record the adsorbed amount in the slit as a function of the (dimensionless) chemical potential (μ = ln φ^b^) of the liquid component. One complication arises because in layers *z* = 1,…, *D*, the bulk volume fraction may not be reached and we cannot simply “pick up” this value from the profiles. As explained in section S1 of Supporting Information File 1, the SCF protocol gives (as output) the volume fraction of a reference system that is in equilibrium with the molecules in the slit. This reference value is used to compute the isotherms. In Figure 5A we present an example for a slit distance *D* = 10 (in lattice units) and our default interaction parameters χ = 2.2 and χ_S_ = −0.3. Recall that under these conditions the surfaces are preferentially solvated by the liquid, and θ_Y_ is 68°. In such situations there is a large van der Waals loop in the adsorption isotherm (cf. Figure 5A). The Maxwell construction can be used to find where, in equilibrium, the step in the isotherm should take place. In line with the hydrophilic character of the surfaces we find the step in the sub-saturated region. In Figure 5A the grey vertical line represents the bulk binodal value. The step takes place at a lower chemical potential (local binodal) than that corresponding to the bulk binodal. We define Δµ as the difference in ln φ^b^ between the local and bulk binodals as indicated in Figure 5A.

It is important to consider the isotherm in slightly more detail. After the jump in the isotherm the adsorbed amount only marginally increases further: the isotherm continues into the supersaturated region and as the system increases the bulk volume fraction, in the limit of very high concentrations the excess should go down again to become zero in the limit of φ^b^ to 1.

The most interesting feature of the isotherm is the presence of a loop. Associated to the loop there are two spinodal points. These spinodal points are located at the turning points of the isotherm. There are two regions of metastability, namely between the local binodal and the first turning point. This spinodal point is found in the region of supersaturation. The other metastable region is found between the turning point in the sub-saturated region and the local binodal in the top region of the isotherm. In the isotherm the metastable branches are indicated by dashed line parts. When in the absence of strong fluctuations the bulk volume fraction is increased, the slit may not necessarily change its contents at the local binodal, but instead remains dry up to – in the extreme case – the spinodal point is reached, and the slit is filled with liquid following the dashed line. Inversely, when the slit is wet, and the bulk concentration is reduced, the drying does not necessarily take place at the local binodal, but enter the other metastable branch. Again the drying must take place before or at the lower spinodal point (following the other dashed line). Hence in dynamical situations a hysteresis loop may be followed where the steps at the spinodal are indicated by the vertical dotted lines. The spinodal points have important roles in the advancing or receding contact line calculations (see below). Even though the van der Waals loop in the isotherm is due to the mean-field approximation, it is found that in real life experiments the system also may be trapped in metastable states very much alike those found in the mean-field model.

In Figure 5B we report that the difference between the local and the bulk binodal in confined spaces is a function of the affinity of the solvent for the substrate. When χ_S_ is more negative the Δµ increases to more negative values. Indeed when χ_S_ > 0, that is for hydrophobic surfaces, the local binodal occurs at supersaturated solutions. With increasing *D* the local binodal shifts towards the bulk binodal.

### Curved L/V interfaces: Kelvin and Laplace

Macroscopic droplets (with negligible curvature) cannot be generated using our method. As the system size is limited, our drops have L/V interfaces that are typically strongly curved. The thermodynamics of curved interfaces is well understood, but there are several complications. One of the issues is that the location of the interface is somewhat arbitrary. On top of this the interfacial tension in curved interfaces cannot uniquely be computed. It depends on the notion of the position of the interface. There exists a choice of the position of the interface, the so-called surface of tension, for which a small notional change of the radius does not influence the value of the surface tension. For this special case the Laplace equation simplifies to Equation 3, and the value of the interfacial tension does not deviate much from the planar value.

From the Laplace equation we know that in droplets with curved L/V interfaces there is a Laplace pressure. As a consequence the chemical potential of the liquid in a drop is at a higher chemical potential compared to systems with planar interfaces. The increased chemical potential is reflected in the oversaturation of water in the vapour phase; a phenomenon named after Kelvin. With oversaturation of the system, which necessarily occurs in our calculations due to the finite size of our droplets, one will invariably get closer to the spinodal point of the capillary condensation process. Hence, oversaturation may trigger the filling of confined regions by the liquid. Small droplets cause a stronger oversaturation than larger droplets and the presence of small droplets may result in a spontaneous filling of the voids by capillary condensation when this may not yet occur for larger drops.

The radius specified by the surface of tension *R*_SOT_ coincides with the visual inspection of where the interface is for many systems. Below we therefore do not exactly determine the exact *R*_SOT_ and use the Ansatz that the interface position is where the solvent volume fraction hits the value φ = 0.5.

### Three-gradient regular solution model

Let us next extend the regular solution theory to model liquid drops at a complex surface topology. We consider a three-gradient coordinate system **r** = (*x*, *y*, *z*) with *x* = 1, 2,…, *M_x_*, *y* = 1, 2,…, *M_y_* and *z* = 1, 2, …, *M_z_*. In contrast to the one-gradient systems where the surfaces were treated through the boundary conditions, in three gradient models it is more natural that the surface component S will occupy lattice sites within the specified volume. Hence, we will specify all the lattice sites within the system: the volume fraction of S is unity and the remainder of the lattice sites are filled by the liquid and vapour components in the usual way. The regular solution free energy is straightforwardly generalised and both interactions between L and V as well as with the surface component S are accounted for:

[13]
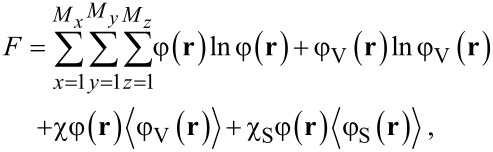


wherein the angular brackets indicate that the free energy accounts for the “curvature” information in three directions. The lattice implementation is simply:

[14]
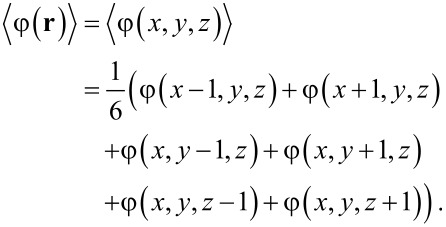


Mirror-like, no-gradient boundary conditions are implemented in boundary layers in the system. This is implemented by setting φ(0, *y*, *z*) = φ(1, *y*, *z*), φ(*M_x_* + 1, *y*, *z*) = φ(*M_x_*, *y*, *z*), and similarly for the other boundaries in *y*- and *z*-directions. Using these boundary conditions it is possible to consider a representative part of the surface, while keeping the computation times and system volumes to a minimum.

In the calculations there are no assumptions regarding the effects of the line tension. The model fully accounts for these effects, but the line tension contributions in our systems were not explicitly extracted. The most important reason why we did not do so is that the line tension cannot be uniquely extracted from the overall grand potential, because a choice for the position of the three-phase contact line is required.

### Specifying the inverse opal

**Figure 6:**
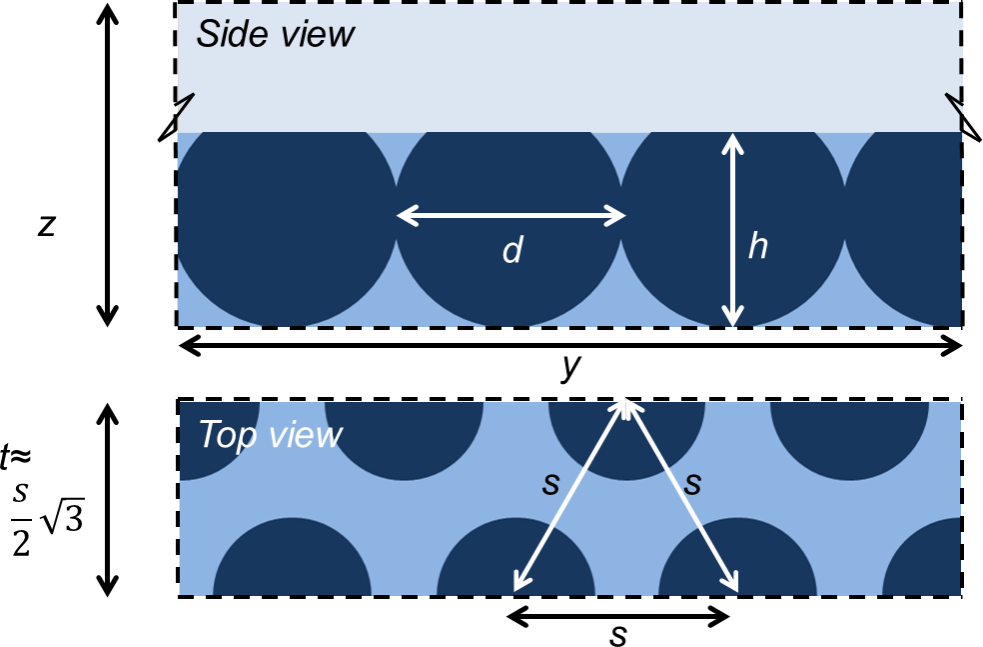
Schematic side view (*z*,*y*)-plane and top view (*x*,*y*)-plane of an inverse opal with two rows of *n* = 3.5 cavities in a staggered packing (3.5 cavity volumes are in the system, four cavity positions are required). The distance between two (*m* = 2) rows is *t*. For a hexagonal packing the two rows are displaced with respect to each other by a value 
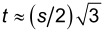
, where *s* is the distance between the cavities. On a lattice *t* must be an integer. Reflecting boundaries are applied in all directions.

The regular solution free energy of Equation 13 still requires detailed information on the distribution of the solid material S in the inverse opal. The idea is to consider a representative piece of a substrate that contains spherical cavities in a specified arrangement (i.e., crystalline ordering with close to hexagonal or square packing symmetries). The cut-off height controls the opening of the cavities as shown in the example of Figure 6. The parameters that control the surface topology are listed in Table 1. Below we will use the parameter *c* = *h*/*d*, which is a fraction at which the cavities were cut and Φ_S_ is the fraction of the “top” of the surface that is solid.

**Table 1:** Parameters that determine the structure of the inverse opal. Even and odd rows of cavities are displaced by half the distance between the cavities in a row, that is, by *s*/2. All quantities are given in lattice units, that is in values of *b*. Below also the cut-off ratio *c* = *h*/*d* (to specify which part of the cavity is cut-off) and Φ_S_ (the fraction of the top of the surface that is solid) is used.

symbol	parameter

*D*	diameter of a cavity (integer > 0)
*S*	spacing between two cavities in a row (integer of order *d*)
*T*	distance between the two rows (integer of order *d*)
*N*	‘number’ of cavities in a row (> 0) may also be non-integer
*m*	number of rows (integer > 1)
*h*	cut-off height of the solid phase (integer > 0)

The parameters of Table 1 completely specify how many cavities are present in the computation volume. Let the cavities be numbered by *i* = 1, 2, 3,…, *N*_c_. In the example of Figure 6 there are two rows of 3.5 cavities and thus we need *i* = 1,…, 8 cavity positions. The input parameters thus specify the coordinates of the cavities {**r**_1_, **r**_2_,…,**r***_i_*,…,**r***_N_*_c_}. The cavities in the inverse opal are placed in rows along the *y*-direction, the direction perpendicular to the rows is the *x*-direction and the cavities are positioned at the lowest *z*-values possible, that is for all cavities *i*, *r_iz_* = *d*/2 (cavity radius).

The solid phase extends up to a height *z* = *h* where *h* is the cut-off height. The solution above the inverse opal starts at a height *z* > *h*. The first and last row in the *y*-direction have their centres on the boundary that is at *y* = 1/2 and *y* = *M_y_* + 1/2, respectively. This implies that the system size in the *y*-direction is *M_y_* = (m − 1)*t*, where *t* is the distance between two rows and *m* is the number of rows. The system size in the *x*-direction is given by *M_x_* = *ns*, with *n* the number of cavities in a row (when half the cavity is in the system the cavity counts by 0.5), and *s* the distance between cavities in a row. In the example of Figure 6, *M_x_* = 3.5*s*. The system size in the *z*-direction should exceed *h* sufficiently so that a sessile drop can be on the substrate. In the first row the first cavity is by default with its centre at *x* = 1/2, that is, at the lower boundary. The first cavity in the second row is positioned at *x* = (*s* + 1)/2, et cetera.

All non-zero surface densities can now be computed. When for a coordinate **r**′ the distance to all of the coordinates {**r**_1_, **r**_2_,…,**r***_i_*,…,**r***_N_*_c_} is larger than the radius of a cavity, i.e., *d*/2 and when the *z*-value is less or equal to *h*, we set φ_S_(**r**′) = 1 and φ_S_(**r**′) = 0, otherwise:

[15]
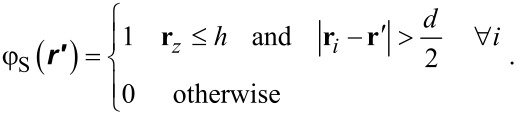


This solid distribution is fixed during the free energy optimisation. Of course only the coordinates that are not taken up by S can be filled with L or V. On the “top” of the solid phase it is of interest to know the fraction of sites occupied by S. These are easily evaluated by

[16]
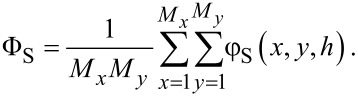


Analytical estimates of Equation 16 are given in section S2 of Supporting Information File 1. The cut-off height *h* normalised by the particle diameter *d* will be referred to by *c*:

[17]
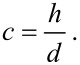


Below we will be interested in hexagonally ordered cavities. For an optimal hexagonal packing, the distance *s* between the particles along a row and the distance *t* between the rows, should obey 
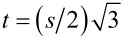
. However, on the lattice only integer values are allowed. Rounding to closest integer values must be implemented. For some values of the particle distances *s* there is a reasonable value of *t*, for other distances the error is relatively large. Only values of *s* which require rounding errors below 0.15 for the corresponding *t* value are used.

The parameters in Table 1 can be used to generate a large variety of inverse opal structures. As long as *s* > *d*, we have the situation that the cavities are isolated. However, the cavities may become interconnected when *s* < *d.* In experimental situations such overlap of cavities may occur, and then there are usually small openings connecting the cavities. This is why this particular parameter setting is allowed.

### Example 1: liquid condensation in a weakly hydrophilic face centred square inverse opal

**Figure 7:**
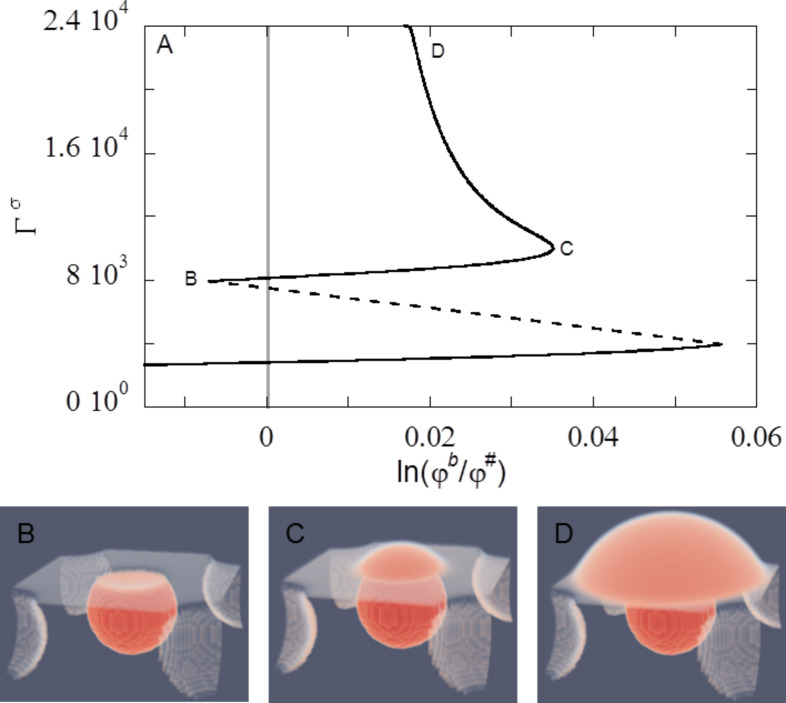
Liquid condensation in hydrophilic inverse opal (*d* = 31, *s* = 88, *t* = 44, *m* = 3, *n* = 1, *h* = 25 (*c* = 0.8)). A) Excess adsorbed amount of the liquid component in the system as a function of the volume fraction of the liquid normalised by the binodal value in lin–ln coordinates. The dashed part represents not the true part of the isotherm but rather connects two states, before the condensation of a pocket and after the condensation. The dotted, vertical line is at the bulk binodal φ^#^. The labels along the isotherm refer to the snapshot colour-coded density distributions given in panels B, C, D. Red is the high-density liquid phase, white is the low-density (gas) phase.

We first consider a simple inverse opal structure that has cavities with a diameter *d* = 31. The distance *s* between the cavities is set to 88, and the distance between the rows *t* = 44. This implies a face-centred square arrangement of the cavities. The number of cavities in a row is *n* = 1, whereas the number of rows is set to 3. As can be seen from Figure 7, this setting generates an equal box size in *x*- and *y*-directions. As the distance between the cavities exceeds the cavity diameter, we have isolated pockets. The surface interaction is set to a slightly hydrophilic value χ_S_ = −0.3.

To compute the adsorption isotherm we start with a low amount of liquid in the system and then increase this amount step by step. In the calculations the outcome of a given calculation serves as an initial guess for the subsequent calculation. This is why a system can be trapped in metastable states, similar to the experimental counterparts.

In Figure 7A we present the adsorbed amount of the liquid component Γ^σ^ = Γ − *V*′φ^b^, where *V*′ is the volume available for the L and V components, which is *V*′ = *M_x_M_y_M_z_* − Σ_r_φ_S_(**r**), as a function of the volume fraction of the liquid component in the bulk. Here we did not normalise with respect to the available surface area and thus this amount is proportional to the surface area. The bulk volume fraction is normalised by the bulk binodal value. The curve is plotted in lin–ln coordinates. Upon an increase in the amount of liquid component in the system Γ, first the vapour phase is gradually saturated with liquid and the adsorbed excess remains modest: At the surface a gaseous adsorption layer develops. Then, upon further increase of the amount, the binodal value is crossed and the system enters the super-saturation regime. The liquid film remains homogeneous along the surface, until Γ^σ^ ≈ 4000, then a first-order jump in the isotherm takes place (cf. Figure 7A, dotted line). As can be seen in Figure 7B, at this stage a droplet formed in the confined space of the cavity and the curvature on the L/V interface in the opening is concave, resulting in a negative Laplace pressure inside the droplet. Since the chemical potential of the liquid molecules should be the same everywhere, this means that the vapour phase is under-saturated (see Figure 7A).

The volume fraction of liquid in the bulk φ^b^ increases as more liquid is added and passes the binodal value φ*^#^* again. At this binodal point there is no under- or oversaturation, hence no curvature of the L/V interface at the opening (not shown). Additional liquid that is added to the droplet induces a convex curvature on top of the droplet, and the bulk oversaturates up to C in Figure 7A. The value of Γ^σ^ at this point is approximately 10000, and consists of a thin film (Γ^σ^ ≈ 4000) and the macroscopic droplet (Γ^σ^ ≈ 6000). The volume of a sphere with *d* = 31 is about 15600. Given that the density difference between the liquid-rich and vapour-rich phase is about 0.5, and that part of the thin liquid film becomes part of the macroscopic droplet, the valur of Γ^σ^ found at point C is in agreement with what is expected for a cavity with diameter *d* = 31.

Additional liquid is subsequently used not to fill the other cavities, but to increase the volume of the existing droplet (Figure 7D) and the droplet starts to spread on the substrate. Below we will follow this process in a slightly different geometry. The oversaturation needed for capillary condensation to occur for the other cavities is in this case not reached. This means that for this inverse opal, with *d* = 31, once condensation has taken place, a droplet grows on top of the filled cavity and the remaining cavities are not filled via capillary condensation, but typically rather fill once the central droplet spreads over and on top of the other cavities (not shown).

Most inverse opals in experiments, which are fabricated using sacrificial particles, have cavities of hundreds of nanometres or more [3,10,42–44]. These sizes are much larger than the cavities considered in the current calculations. Smaller cavities can fill more easily via capillary condensation (cf. Figure 5B). Since the impregnating wetting state is not observed even for such small cavity sizes as used in Figure 7, we conclude that the impregnating wetting state due to capillary condensation is not likely to develop for practical inverse opals which are marginally hydrophilic.

In this surface structure, the cavities are not connected, whereas for some experimentally fabricated inverse opals all cavities may be interconnected via a small opening. Since the curvature of the droplet should be constant, the curvature of the liquid–vapour interface at the small opening is the same as for the rest of the macroscopic droplet [13], and hence the high curvature needed for the next cavity to be wetted via this opening, is not reached. Therefore, a cavity that is filled with liquid will wet the next cavity via the larger opening at the top, rather than through this small hole (not shown).

### Droplets on top of the hexagonally ordered inverse opal

In the remainder of this paper we will focus on close-to-hexagonally packed cavities. In principle one can force a water-front to move along such a surface in an arbitrary direction. Here we focus on just one of the possible directions.

We consider solvent fronts along the *x*-direction, which spread by increasing the volume of the droplet, in the *y*-direction. Recalling that mirror-like boundary conditions are implemented in *x*-, *y*- and *z*-directions, in this scenario it suffices to have just two rows of cavities, that is *m* = 2. The system is much larger in the *y*-direction and we consider *n* cavities with a spacing *s*. For a hexagonal packing of cavities the distance *s* and the spacing *t* between rows are interconnected and we will mention just one of these parameters. The number of cavities that are considered in the *y*-direction is taken sufficiently large so that there are no boundary effects. The surface is thus sufficiently specified by mentioning the cavity diameter *d*, the distance between the cavities *s* and the cut-off height *h*, or equivalently *c* = *h*/*d*. Typically, we will initiate the calculation by means of some initial guess of the SCF. protocol such that a droplet develops with its symmetry plane along the *y* = 1/2 boundary.

### Example 2: Advancing and receding drop fronts on a slightly hydrophilic hexagonally packed inverse opal

**Figure 8:**
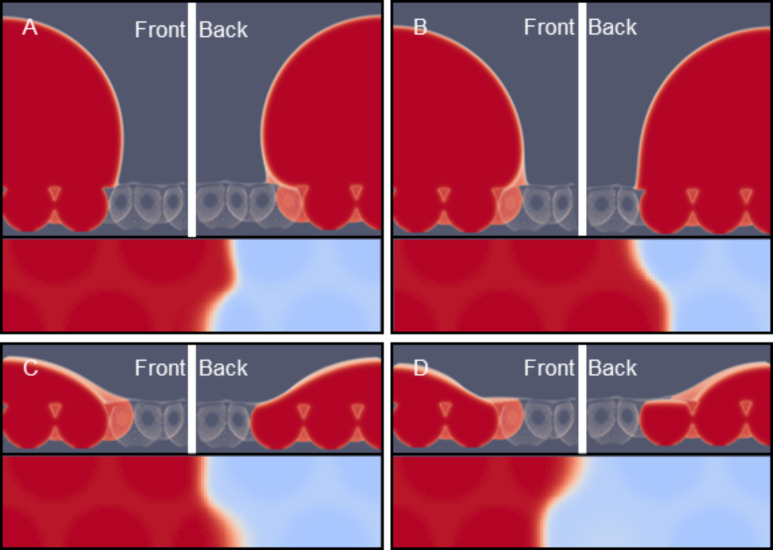
Four examples of droplets on an inverse opal with *d* = 31, *s* = 30, *c* = 0.80, *n* = 3.5, and χ_S_ = −0.3. A,B) Two advancing water fronts with relatively high contact angles; C,D) two receding water fronts with low contact angles. For each panel the left (*z*, +*y*), right (*z*, −*y*) and bottom images (*x*, *y*) are for different view-points, that is, the left image is taken from the front, the right image is taken from the back, the bottom image is the top-view. Colour coding: Red is high-density liquid. Blue is low-density gas. White is intermediate density. The snapshots are taken from the calculations of Figure 9.

**Figure 9:**
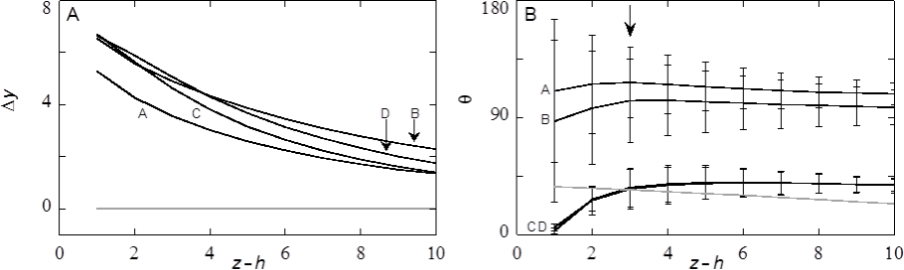
Examples of drop characteristics. A) The standard deviation (measured in *x*-direction) of the position of the interface Δ*y*′ as a function of the height above the substrate *z* − *h*. The horizontal grey line represents the result on a smooth surface. B) The angle of the liquid/vapour front in the (*z*,*y*)-plane, θ(*z*) together with the standard deviation of the angle measured in the *x*-direction plotted as “error” bars. The grey curve is the contact angle of a similarly sized droplet on a smooth surface. The labels A–D correspond to the snapshots A–D in Figure 8: A,B advancing contact liquid front; C,D are receding liquid fronts.

In Figure 8 we give representative examples of planar solvent droplets with their solvent front (on average) along the *x*-direction. The drops sit with their symmetry plane at *y* = 1/2. The inverse opal is characterised by the cavity diameter *d =* 31, the spacing between the cavities in the *y-*direction *s =* 30*,* the cut-off fraction *c =* 0.80*,* and the number of cavities in the *y*-direction, *n =* 3.5*.* The surface is slightly hydrophilic (χ_S_ = −0.3). The cavities directly under the macroscopic water front are filled with liquid, while the other cavities remain empty. The water front is thus in the Wenzel wetting state (Figure 1). Notice that an additional cavity is filled going from A to B. Such an event gives discontinuities as discussed below. In B there is slightly more liquid in the system than in A, but the height of the drop in A is more than that in B. The liquid in the cavity is noticed as a volume reduction in the drop. The panels A and B are taken as examples of the droplet shape in a series of calculations for which the droplet volume was increased (see also below in Figure 9).We refer to these as advancing front lines. The other two panels (C and D) are taken for the situation that the drop volume was decreased and we refer to this situation as receding front lines. Three different view positions of the same drop are given in this figure to illustrate the features that present themselves in advancing and receding cases. From the top-view perspective, we see that the solvent front is not straight. It curves along the cavity openings and the exact shape of the front strongly “fluctuates” depending on the exact value of the droplet volume.

In one of the cases the front is at lower *y*-values at low *x*-values and in the other case it is inversed, the lowest *y*-value is at a high *x*-values. In the four cases shown in Figure 8 we see that the absolute value of differences in the *y*-position does not depend much on the advancing or receding modes.

As the three-phase contact line is curved, necessarily the contact angle must vary as well. The contact angles are best viewed from the side. The top graphs in Figure 8 are images taken from a “front” or “back” view point. We present both of these to illustrate that the shape in the (*z*,*y*)-plane depends slightly on the *x*-coordinate. Clearly, there is a huge difference in the contact angle between the advancing fronts (very high angles) and the receding fronts (very low angles). Furthermore, as can be seen in panel D, the receding droplet remains pinned on top of the liquid-filled cavity, resulting in a longer contact line as compared to advancing droplets.

For a given snapshot we can evaluate the contact angle θ(*x,z*) in the (*z*,*y*)-plane by estimating by interpolation the position *y*′ of the interface, where the φ(*x*, *y*′, *z*) = 0.5. In other words, the *y*′-position of the liquid/vapour interface depends both on *x* and *z*: *y*′ = *y*′(*x*, *z*). Then the local contact angle of the solvent front is a function of both *x* and *z*:

[18]
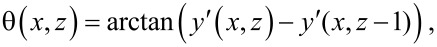


which implies that the contact angle can only be computed for *z* > 2. The average angle at a height *z* is found by averaging along the *x*-direction:

[19]
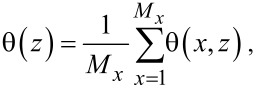


while the standard deviation Δθ(*z*) measured in the *x*-direction is given by

[20]
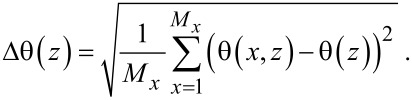


Similarly, the position of the interface *y*′(*x*,*z*), the average position of the interface *y*′(*z*) and the standard deviation Δ*y*′(*z*) are straightforwardly recorded.

In Figure 9A we plot the standard deviations Δ*y*′(*z*) as a function of *z* − *h* (height above the substrate), and in Figure 9B θ(*z*) together with the fluctuation in the angle Δθ(*z*) as “error” bars as a function of *z* − *h* for the four droplets already shown in Figure 8 is plotted.

It is natural to expect that when the drop characteristics are considered further away from the surface that the influence of the surface is gradually reduced. This is why the Δ*y*′(*z*) is a decreasing function of *z*. Again, as noticed already from the snapshots, the value of Δ*y*′(*z*) does not depend much on the advancing or receding modes of wetting. That is why the four curves in Figure 9A are nearly the same. We refrain from trying to provide further comments about the differences. Similarly, far from the surface the angles become independent on *x*. That is why in Figure 9B the “error” bars diminish in size when *z* − *h* is increased. Indeed very close to the substrate *z* − *h* < 4 the fluctuations are very large, that is about 50% of the value of θ.

We already noticed that the advancing contact angles are much larger than the receding ones. Figure 9B gives the numerical values more accurately: The advancing angles are on average larger than 90°, whereas the receding angles are about 45°, very close to the angles found for the unstructured surface (grey line). Interestingly, the average contact angle in the advancing mode can go through a small local maximum at a height *z* − *h* = 3. Such an effect hints to the presence of a foot on the droplets. However, at this height the fluctuations are large and we are hesitant not to over-interpret the results.

It is clear that, if we want to compare droplets and see trends, we need to reduce the outcome of the computations. That is why from hereon we will focus on the properties of the droplets on a height of *z* − *h* = 3 (as indicated by the arrow in Figure 9). At this height above the substrate the “foot” is not disturbing too much, while the structure of the surface is still well noticeable. We thus define (if not mentioned otherwise) the fluctuations of the liquid front measured as the standard deviation along the *x*-direction as Δ*y* = Δ*y*′(*h* + 3), and the average contact angle of the drop θ = θ(*h* + 3), as well as the fluctuations Δθ = Δθ(*h* + 3).

**Figure 10:**
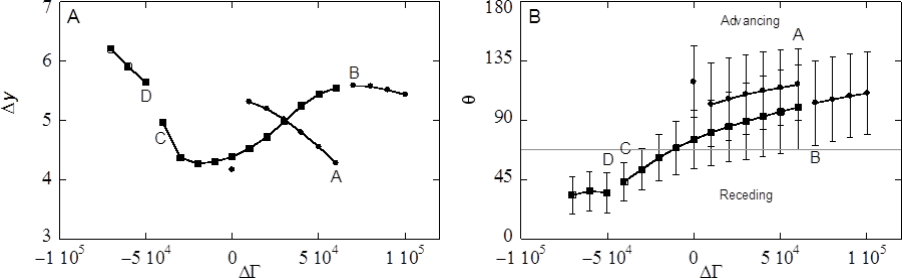
Examples of structural features of advancing and receding drop fronts. A) The fluctuations of the position of the liquid front along the *x*-direction at a height *z* = *h* + 3 as a function of the ΔΓ of the droplet component with an initial amount Γ = 3·10^6^. B) The corresponding average contact angle θ (measured at a height *z* = *h* + 3) and the standard deviation (plotted as error bars). The advancing liquid front is given by the solid sphere data points, the receding ones in solid square data points. The lines are to guide the eye. A discontinuity in the line represents a jump-wise change of the drops on the substrate. The labels A–D correspond to the snapshots of Figure 8 and the structural data of Figure 9. A and B are along the advancing branch, whereas C and D are taken from the receding drop fronts. Parameters are similar as in Figure 8 and Figure 9.

The calculations of Figure 8 and Figure 9 were started with an initial amount of liquid Γ *=* 3·10^6^. More liquid is added to the system and these molecules are consumed by the drop. Hence the drop volume increased. Typically we performed ten liquid addition steps and for each of these new profiles are calculated to obtain an advancing angle. The amount of the liquid is subsequently stepwise decreased to check for hysteresis and to obtain a receding angle. The structural properties of the drops are recorded during this cycle and the results were collected in Figure 10.

The starting point of the calculations is not extremely well defined in terms of advancing or receding states. The initial guess takes the system in this case close to an advancing situation: the contact angle θ is rather high. Typically this initial drop is disregarded from our averaging (see below). Upon stepwise increase of the drop volume (closed spheres) Δ*y* decreases from 5.5 to 4.0 while the contact angle θ increases gradually until point A is reached. The contact line did not move upon adding the liquid: The contact line is arrested as the contact line cannot be placed on top of a (water filled) cavity [45]. Then with a small increase the system jumps from A to B. Above we saw that in this event one extra cavity is filled with liquid. At this event the contact line de-pins jump-like in an event that may be referred to as a de-pinning transition [19]. In Figure 8A,B we see that the three-phase contact line has the opposite curvature in the *x*-direction. At this de-pinning event the average contact angle jumps downward to θ ≈ 90° (cf. Figure 10B) and Δ*y* jump-like increases from 4.0 to 5.5 (cf. Figure 10A). The contact angle close to the surface and directly in front of the cavity that fills up, switches thereby from θ > 90° to θ ≈ 90° (cf. Figure 8A,B). These increased contact angles with respect to θ_Y_ (68° for χ_S_ = −0.3) are hereby found for a hydrophilic surface without air entrapment. The system may experience more of such events when more and more liquid is added. After another four additions of volume we were close to the initial condition and the advancing contact line calculations were stopped.

For receding water fronts (Figure 8C,D), computed by taking liquid out of the system (the squares in Figure 10), we first retrace a part of the advancing curve, that is, the four latest volume additions were undone and the same results were recovered. However, as soon as the volume of the drop is decreased compared to point B we follow a different route. We do not jump to point A, but rather follow the trend downward for the average contact angle θ (cf. Figure 10B) while also the fluctuations Δ*y* decrease. Close to the initial volume the contact angle is found to be close to the value on the smooth surface θ = θ_Y_. At this point the curvature of the dependence Δ*y*(ΔΓ) changes. Further reduction of the volume of the drop leads to a local minimum of Δ*y*, while the contact angle drops significantly below the value of θ_Y_. Then point C is reached (cf. Figure 8C). Now the receding front is pinned on top of a liquid filled cavity. The low contact angle on top of the cavity, clearly visible for the right-hand site of droplet Figure 8C, is explained by the fact that the liquid wets a surface of the same material (namely the liquid in the cavity). During receding, the water front is thus pinned at the liquid/liquid surface with a local θ of 0°. The cavity remains filled after the droplet has retracted from the cavity (see cavity on the right-hand site of droplet D) which occurs once again step-wise. During this de-pinning step the three-phase contact line rearranges its curvature again (cf. Figure 8C,D). At this de-pinning transition the value of Δ*y* increases jump-like, while the average contact angle decreases somewhat. Upon further reduction of the drop volume the Δ*y* increases further while the contact angles remain low.

The traces of Figure 10A,B imply a hysteresis: The curves for adding and reducing volume only overlap when no de-pinning transition has occurred in between the addition or removal steps. The contact line of the advancing droplet experiences a surface consisting of solid and vapour, whereas the contact line of the receding angle experiences a surface consisting of solid and liquid. The true receding angle is only visible for values of ΔΓ between −5·10^4^ and −7·10^4^ (last three data points) and is about 35°. If the surface can be regarded as consisting of a solid with a liquid, then the contact angle can be calculated using [15]

[21]
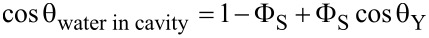


with the fraction of solid on the top of the substrate Φ_S_ = 0.43 (for *c* = 0.8). The contact angle according to this calculation (40°) is in agreement with the contact angle we find here. For these last droplets, high values of Δ*y* are found. The contact line is in these cases pinned on the farther edge of a water-filled cavity (see Figure 8D).

### Trends in the shape of advancing water fronts

In the remainder of this paper we will focus on advancing contact angles. Referring once again to the results of Figure 10B, we typically initiated calculations with the drop near the lower boundary on the *y*-axis in such a way that the resulting drop shape assumes properties of an advancing one. Then, as in Figure 10B ten subsequent increases of the amount of the liquid component were implemented. The average contact angle θ (along the *x*-direction and at *z* = *h* + 3) were again averaged over these ten droplets to obtain 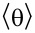. Typically one or more de-pinning events were accepted in this averaging. Recall, that in the advancing branch the contact angle in a de-pinning event changes only slightly. The standard deviations were averaged similarly.

### Effect of cavity size *d*

The size of the inverse opals in the calculations, which is linked to experimental sizes by the width of the interface, invariably is much smaller than the size of inverse opals used in experiments. To study the effect of size of the structures on the (double) averaged advancing contact angle 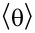, droplets on inverse opals with cavity diameters ranging from 9 to 52 have been recorded. The trend observed for this series of sizes, can be extrapolated to even bigger sizes without the need to calculate those. In these calculations the spacing between the cavities was set to *d* − 1, that is, the cavities were slightly overlapping so that a small hole connects the cavities. The cut-off fraction is kept at *c* = 0.8.

**Figure 11:**
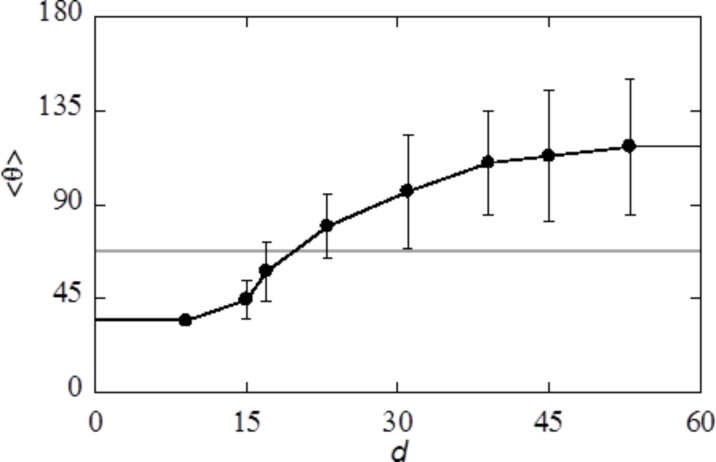
Advancing averaged contact angle 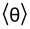 and standard deviation as a function of the cavity diameter *d* (in lattice units) for a hexagonally ordered inverse opal with cut-off fraction *c* = 0.8, distance between cavities *s* = *d* − 1 and slightly hydrophilic surface properties χ_S_ = −0.3. The grey line is contact angle θ_Y_ on a smooth surface.

As shown in Figure 11, the 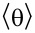 is an increasing function of the cavity diameter *d*. For *d* > 40 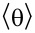 reaches a plateau. The levelling off hence implies that it is not necessary to increase the cavity sizes even more to reach the experimental limits.

The fact that 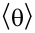 can increase above θ_Y_ is attributed to the pinning of the contact line around the cavities. For very small cavities we observed that the average angle can be smaller than θ_Y_ (grey horizontal line in Figure 11). Small cavity sizes are similar to the small confinements *D* used in the one-gradient slits of Figure 5. From these slit calculations we know that small values of *D* need only a small oversaturation to fill the slit with the liquid. Similarly, very small cavities can easily be filled with the liquid. We refer to this situation as the impregnated wetting state (Figure 1). Indeed the inverse opal with *d* < 10 are in the impregnated state and it is natural to expect that for this case 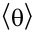 < θ_Y_: the effective top surface in front of the water front consists of the solid with patches of liquid, just as is the case for a receding droplet. The advancing contact angle 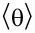 found for these structures (see Figure 11) corresponds to the receding contact angles presented in Figure 10B.

For the structure with *d* = 15 the situation is rather complex. It appears that some, but not all, cavities in front of the droplet are filled with liquid. In the process of advancing the liquid front, we add more and more of the liquid. As soon as an additional cavity is filled, the volume for the droplet decreases and this reduces the curvature and the corresponding oversaturation in the vapour phase. This decrease in oversaturation after filling subsequent cavities prevents other cavities to fill up. The number of cavities filled in front of the water front, depends on the size of the droplets on top of the inverse opal: the smaller the droplet, the higher the curvature, thus the more oversaturation. The more cavities are filled, the lower is the advancing contact angle 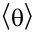 as the surface becomes effectively hydrophilic. The droplet size dependence will imply small changes in the dependence of 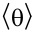 as a function of *d* in Figure 11. For the drop sizes used to compute Figure 11 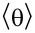 = θ_Y_ occurs approximately at *d* = 20.

For the inverse opals with d ≥ 20 only cavities directly under the droplet are filled (Wenzel wetting state). The water front encounters the same fraction of flat solid top surface, Φ_S_ for all cavity sizes, but the increase in 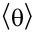 with *d* depends on the strength of the pinning effects and this allows 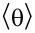 to increase with *d* above θ_Y_.

### Effect of the S/L interaction parameter χ_S_

Let us next focus on the interaction of the liquid with the substrate via the S/L interaction parameter χ_S_. The more negative this parameter the more hydrophilic (solvophilic) the surface is. When the χ_S_ is positive we may refer to the surface as hydrophobic (solvophobic). We select for this study hexagonally ordered inverse opals with a cavity size *d* = 39. As shown in Figure 11 such cavity sizes give wetting features in the plateau region where the size dependence was essentially lost. We consider here the case that the cut-off fraction is *c* = 0.9. This *c*-value is chosen to mimic the inverse opal in [10]. The distance between the cavities was set to *s* = *d* − 1 (cavities are connected to each other by small openings).

**Figure 12:**
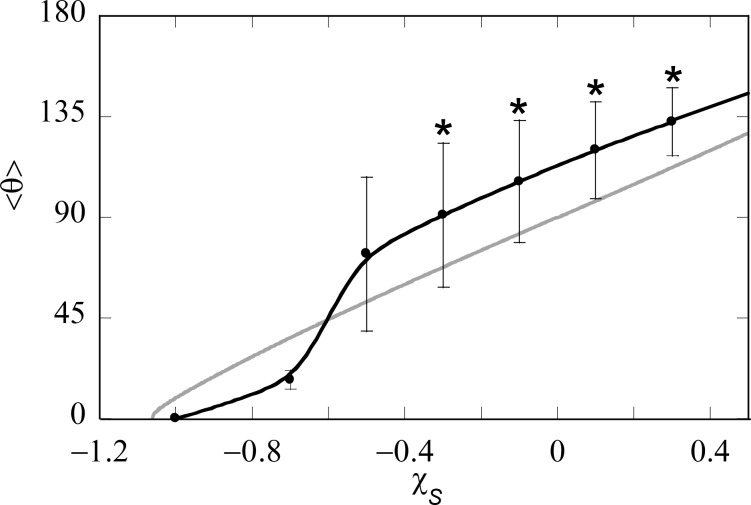
Advancing contact angle 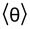 and the corresponding fluctuations (measured along the *x*-direction) indicated as error bars, as a function of the affinity of the solvent for the substrate phase χ_S_ for a hexagonal inverse opal with cavity sizes *d* = 39, cut-off fraction *c* = 0.9 and spacing between the cavities *s* = d − 1 = 38. The contact angle on a smooth surface is shown with a grey line (copied from Figure 4). Stars indicate that the cavities under the water front remain empty (Cassie–Baxter wetting state).

In Figure 12 the grey curve represents the contact angle θ_Y_ on the smooth surface as a function of χ_S_ and this result is reproduced from Figure 5 for ease of comparison. The average advancing contact angle 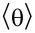 increases with decreasing hydrophilicity (solvophilicity) of the substrate. However, for χ_S_ > −0.6 the advancing contact angle is systematically above θ_Y_, whereas it is systematically below θ_Y_ when χ_S_ < −0.6.

The wetting transition, which is the point for which θ becomes 0, occurs at a value of χ_S_ which is slightly less hydrophilic for the inverse opal as for the smooth surface. In the low contact angle cases all cavities under the droplets are filled (impregnating wetting state). The filled cavities render the surface slightly more hydrophilic than the smooth surface (see Equation 1) and this caused the early wetting transition for the inverse opal as compared to the smooth surface.

The inverse opal remains in the impregnating wetting state for small but finite contact angles χ_S_ = −0.7. In these cases the advancing contact angle is lower than the corresponding θ_Y_. The wetting switches to the Wenzel state by increasing the hydrophilicity further to χ_S_ = −0.5. Now the advancing contact angle is larger than θ_Y_. Eventually, the wetting switches to a Cassie–Baxter wetting state for higher values of χ_S_. These cases are labelled by the asterisk in Figure 12. Again the advancing contact angle is larger than θ_Y_, and the difference 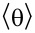 − θ_Y_ is roughly constant, that is, it does not depend whether there is the Wenzel or the Cassie–Baxter state. This result suggests that for the advancing angle, it does not matter whether air is entrapped underneath (observed for χ_S_ ≥ −0.3) the droplet or not (observed for χ_S_ ≥ −0.5). This implies that the increase in observed 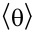 compared to θ_Y_ cannot be explained in terms of wetting state. Rather, pinning of the contact line and the de-pinning transition should be considered. The immobilization of the contact line on the kinks in the surface (as explained in Figure 2) on this hexagonally packed inverse opal induces the contact line to curve and extend (Δ*y* > 0), which is energetically unfavourable. The contact line is even further extended after a de-pinning transition (increase in Δ*y*), indicating that in the stress perpendicular to the surface has increased. However, 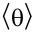 decreased locally to values close to θ on a smooth surface, showing that stress in the vertical direction is released after the transition. The average 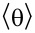 is still higher than θ_Y_ directly after the de-pinning transition. This is caused by the build-up of stress at the other cavity (we consider two rows of cavities).

The interplay between curvature perpendicular to the surface (here discussed in terms of 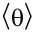) and parallel to the surface (here discussed in terms of Δ*y* of the contact line) results in an overall high 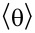. Whether the cavity under the moving water front fills with liquid or remains filled with air depends on χ_S_: Air is entrapped for more hydrophobic materials, but the cavities fill for a more hydrophilic material. The pinned water front on a surface with higher χ_S_ can withstand higher 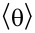 before stress starts to build up and the de-pinning transition occurs.

### Effect of cut-off height *c* at constant spacing *s*

**Figure 13:**
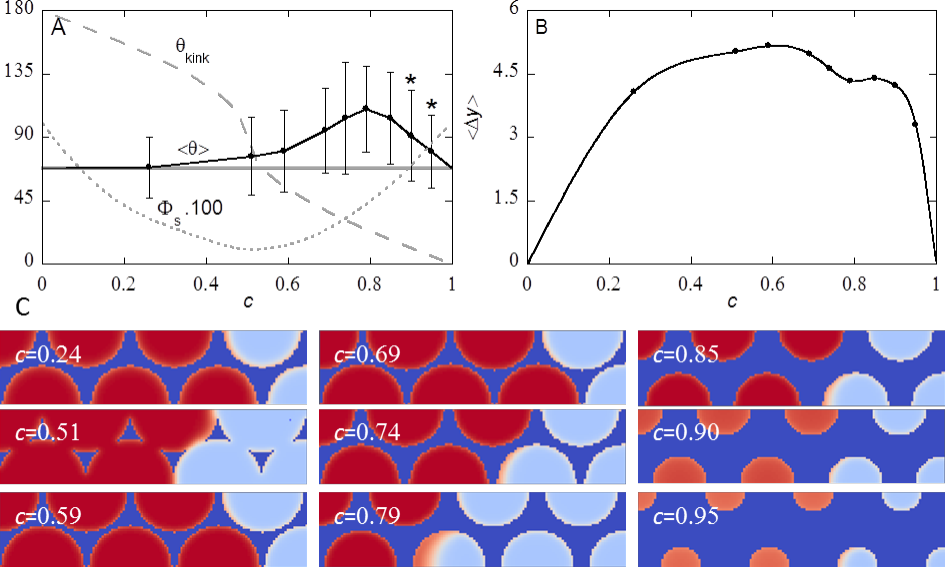
A) Advancing contact angle 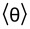 and the corresponding standard deviation as measure of variation in the *x*-direction as function of the cut-off fraction *c*. The horizontal grey line is the contact angle θ_Y_ on a smooth surface, the grey dashed line is the angle of the kink θ_kink_ at different values of *c* and the grey dotted line is Φ_S_∙100. Stars indicate that the cavities under the water front are filled with vapour (Cassie–Baxter wetting state). B) Corresponding average variation in *y*-position 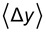 at *z* = *h* + 3 as function of the cut-off fraction *c*. The hexagonally ordered inverse opal has cavity diameter *d* = 39, spacing between the cavities *s* = *d* − 1 =38, and χ_S_ = −0.3. C) The distribution of solid S at the top layer of the substrate, that is φ_S_(*x*, *y*, *h*). The dark blue colour implies density of the solid is unity. The red colour indicates that the opening of the cavity is filled by the solvent. The light blue colour means that the cavity opening is filled with vapour. Orange colour implies that the solvent density is in between the liquid and the vapour. The value of *c* is indicated.

Experimentally one can control the inverse opal structure by the cut-off height *h*, or equivalently to the cut-off fraction *c*. It is of interest to consider the effect of the cut-off height from a computation point of view. We can study this for a fixed spacing *s* between the cavities (in this section) or with a fixed fraction of solid in the top of the substrate Φ_S_ (next section). In both cases we choose slightly hydrophilic substrates with χ_S_ = −0.3. This value of χ_S_ gives θ_Y_ = 68°. Similarly as in the previous paragraph we fix *d* = 39 and *s* = *d* − 1. Obviously the limits *c* = 0 and *c* = 1 are the same as the smooth surface. In between these two limits the surface structure is characterised by a top surface layer with a fraction of Φ_S_ of the sites being the solid. The top layers are given in two-gradient contour plots in Figure 13C. When *c* = 0.5 the amount of S (dark blue colour) is minimal: both in the limits *c* = 0 and *c* = 1 the cross-sections are completely blue (not shown).

Correspondingly, the contact line fluctuations, as monitored by Δ*y*, are averaged over ten “snapshots” while increasing the drop volume. The result is given by 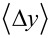. In both limits *c* = 0 and *c* = 1 the surface is ideally smooth and the three-phase contact line will not fluctuate in the *x*-direction. In between these limits 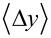 > 0 because the contact line becomes pinned.

The advancing contact angle 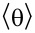 together with the standard deviation of this angle (measured in the *x*-direction) is presented in Figure 13A as a function of the parameter *c* = *h*/*d*. The corresponding fluctuations of the three-phase contact line 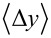 (measured in the *x*-direction at a height *z* = *h* + 3) are given in Figure 13B.

Inspection of Figure 13A shows that by far the most interesting region is for 0.5 < *c* < 1. For *c* < 0.5 the average advancing contact 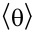 hardly differs from θ_Y_. However, for small values of the cut-off fraction the contact line is already significantly curved (Figure 13B) and the surface structure alters θ(*x*) locally to hydrophobic values θ > 90°. The average 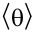 for 0.51 < *c* < 0.95 is higher than θ_Y_ on a smooth surface, though locally, θ(*x*) may be smaller. The variation of θ along the *x*-direction (depicted as vertical line) extends to values θ < 68° for most values of *c*. A maximum in 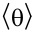 is found at *c* ≈ 0.80, with 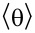 = 110 ± 30°. A local minimum in Δ*y* at this value of *c* is found, which likely is coupled to the need to keep the overall curvature in the drop constant.

Two parameters of the surface structure that are important for contact line pinning are changed when *c* is varied: the fraction flat solid top surface Φ_S_, which is the fraction of flat solid at the top of the surface (dark blue in Figure 13C) and the angle of the kink, θ_kink_ (see section S2 of Supporting Information File 1 for calculation of θ_kink_ and section S3 of Supporting Information File 1 and Equation 16 for the calculation of Φ_S_). Both, Φ_S_·100 and θ_kink_ are plotted in Figure 13A. The value of Φ_S_·100 equals 100 in both limits of *c* and has a minimum at *c* = 0.5. θ_kink_ is 180° at *c* = 0, and decreases to 0 at *c* = 1. The latter parameter is considered important for hydrophilic materials to obtain hydrophobic contact angles. It has been suggested that only for θ_Y_ < θ_kink_ pinning can occur, and a barrier for the water front to enter the cavities is obtained, resulting in air entrapment and the possibility of obtaining higher contact angles θ. However, in our case, θ_kink_ is smaller than θ_Y_ for most values of *c*, and no jump in 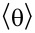 is observed between *c* = 0.51 (θ_kink_ > θ_Y_) and *c* = 0.59 (θ_kink_ < θ_Y_), and no air entrapment is found for 0.59 < *c* < 0.85.

For *c* = 0.26, θ_kink_ is too large for pinning to occur according to the argument presented Figure 2. However, an advancing water front is immobilized at the front of a cavity, and moves in one step to a position in front of the next cavity. Hence, despite of the small θ_kink_ in our calculations a true de-pinning transition is found.

In our case the pinning of the contact line is due to the complex 3D-structure of the surface: the cavities are placed close to each other, and the contact line thus encounters multiple cavities on a short distance. If the water front was not pinned at the front of a cavity, but rather partly filled the cavity, this would result in a larger L–S interface and a longer contact line. This is apparently energetically more unfavourable than pinning the contact line in front of a cavity. Hence, the contact line pinning is governed by the 3D-structure, and not by the simple 1D-argument presented in Figure 2.

When looking at the top surface (Figure 13C) for *c* = 0.26, we see that some cavities on the left are filled (red), while the last 1.5 cavities are not filled (light blue). The last upper cavity is just in front of the contact line. Some liquid (red colour) is present at this value of *z*. Moreover, the situation for *c* = 0.74, which has a similar top surface but a different θ_kink_, is comparable.

For *c* close to 0.5, the substrate is not a continuous structure, but consists of discrete triangular “pillars” (see Figure 13C). Contact line pinning also occurs for discrete shapes [19,46]. Assuming that pinning is mainly found on the top surface in line with observations for *c* = 0.26, the length over which pinning can take place is limited. However, also in this case a de-pinning transition is observed for an advancing water front. Hence the pinning is not limited to the flat part of the top surface (the dark blue surfaces in Figure 13C) for all values of *c*, but pinning occurs over the 3D structure.

Decreasing or increasing *c* from *c* = 0.5 results in a continuous top layer. The contact line can hereby be pinned at the front of every cavity. For higher values of Φ_S_, the contact line is expected to be exclusively located on the top surface (and thus at constant *z*). Higher values of Φ_S_ (thus smaller cavities) also result in a surface that is more similar to a smooth surface.

In Figure 13C, the liquid-filled cavities are shown in red, whereas vapour-filled cavities are light blue. This is observed for all *c* up to *c* = 0.85. However, for *c* = 0.90 and *c* = 0.95, we observe a colour in between red and light blue for cavities under the droplet (left hand side). This suggests that at that height, neither a liquid, nor a vapour phase is present, and thus that the interface is located at that value of *z*.

### Effect of cut-off height *c* at constant Φ_S_

One may argue that the true effect of the cut-off height is seen for cases where *h* (or equivalently *c*) is varied at a fixed amount of S in the top layer of the substrate, that is for fixed value of Φ_S_. To do so, one has to vary the inter-cavity spacing *s* simultaneously when the cut-off height is changed. Note that the true limits *c* = 0 and *c* = 1 are hard to reach with fixed Φ_S_ as it requires odd distances between the cavities.

**Figure 14:**
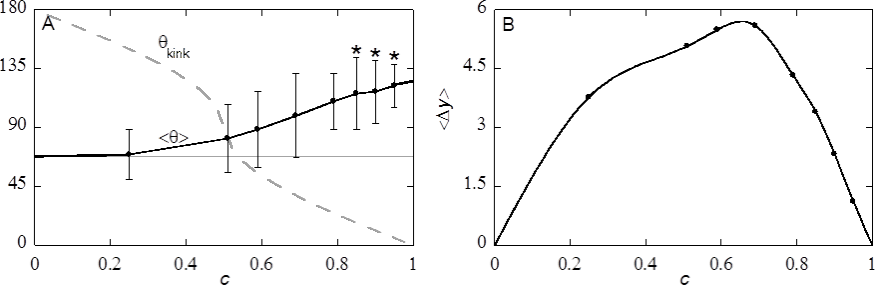
A) The advancing contact angle 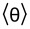 and standard deviation as function of cut-off fraction *c* for fixed value of the solid fraction in the top-layer of the substrate Φ_S_ = 0.43. The grey line is θ_Y_ on a smooth surface and the grey dashed line is the angle of the kink at different *c*. Stars indicate that the cavity under the liquid front are vapour-filled (Cassie–Baxter wetting state). B) Variation in *y*-position 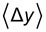 as function of cut-off fraction *c* for *d* = 39 and χ_S_ = −0.3.

The behaviour of the advancing contact angle as well as the contact line fluctuations are recorded for this scenario in Figure 14. Here we have chosen Φ_S_ = 0.43, which corresponds to *c* = 0.79 (near the maximum) of Figure 13. Note that all the top surfaces used in Figure 14 are similar to the result shown in Figure 13C for *c* = 0.79.

It is natural to compare results between Figure 13 and Figure 14. For values of *c* < 0.79, the average contact angles differ very little. In Figure 14 the average angle appears slightly larger. There is one dramatic difference between Figure 13 and Figure 14. Compare, for example, the top surface for *c* = 0.51. While in the case of fixed distance between the cavities *s* there are individual posts with Φ_S_ ≈ 0.1 (Figure 13C), there is a continuous solid phase in Figure 14 as Φ_S_ = 0.43. Nevertheless, the average contact angles were hardly affected.

At *c* > 0.79, 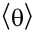 at constant Φ_S_ (Figure 14) keeps growing with the increase in *c*, while at fixed *s* the contact angles decrease again. The decrease in 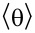 for *c* > 0.79 in the case of constant *s* (Figure 13) can thus be attributed to the top surface: Higher *c* result in smaller cavities and thus in a surface that is more similar to a smooth surface. The increase in 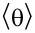 found for increasing *c* at constant Φ_S_ implies that, assuming a constant line tension, a higher θ_kink_ for a hexagonally packed surface structure gives rise to a higher barrier for a de-pinning transition to occur and thus higher average contact angles can be maintained. The higher average contact angles are produced with lower and lower fluctuations of the shape of the contact line. Eventually, at the limit of *c* = 1 the fluctuations must vanish by definition.

Interestingly, it is found that the cavities under the water front remain empty for *c* = 0.85 (and up) at Φ_S_ = 0.43, whereas the cavities were filled for *c* = 0.85 at *s* = 38 (Φ_S_ = 0.55). Bringing the openings of the cavities closer to each other thus prevents water from entering the cavity.

### Summary and Outlook

We have implemented a regular solution lattice model to study the wetting on a structurally complex surface. The interaction between vacancies and liquid is parameterised in a regular solution model by the Flory–Huggins parameter χ. This parameter controls the width of the interface between liquid and vapour. The model allows for a detailed description of the solid phase and the liquid–solid interaction parameter χ_S_ is the only parameter to control the hydrophilic/hydrophobic (solphophilic/solvophobic) character of the substrate. We find very complex and interesting wetting states when this model is applied to hexagonally ordered cavities in an inverse opal. It is found that the three-phase contact line is curved and becomes pinned at the cavity openings. Under the droplet the cavities can either be filled with water (impregnated; Wenzel) or filled with the vapour (Cassie–Baxter). No discontinuity in the contact angle θ is observed for water fronts that are either in the Wenzel state or Cassie–Baxter state, implying that the water front shape is not influenced by air entrapment under the water front, but is rather determined by the surface encountered by the contact line. The pinning of the contact line cannot solely be discussed in terms of the kink that a surface structure makes with respect to the top surface, θ_kink_. Also, for θ_kink_ > θ_Y_, de-pinning transitions are found, and the spacing between the cavities influence whether a cavity under a water front is solvent-filled or vapour-filled. Hence, the full 3D-structure, rather than one parameter (θ_kink_) should be taken into account. We found a large difference between advancing and receding contact angles, which are also attributed to pinning of the three-phase contact line. More specifically, it was found that while the smooth surface has a contact angle much lower than 90° the advancing contact angles in the inverse opal can be much larger than 90°. That is, slightly hydrophilic substrates can give hydrophobic contact angles.

Our method can readily be extended to mimic experimental conditions more closely. An interesting case is the wetting of an inverse opal of polypyrrole [10]. The inverse opal of polypyrrole does not have a smooth top surface. Rather, the top surface showed a positive slope radiating from the position of the sacrificial particle. This more complex surface structure can be implemented easily by a more elaborated way to represent the substrate. Furthermore, polypyrrole is a hydrophilic material. The angle θ_Y_ on a polypyrrole surface was measured to be about 20°. However, the inverse opal was made using sacrificial polystyrene particles. The particles were removed by dissolving them. This removal was expected to be incomplete, and the contact angle θ changed to about 80° for the polypyrrole surface. Polystyrene chains thus must have remained, e.g., in an adsorbed state onto the polypyrrole surface. These adsorbed polymers have a different hydrophilicity, but also changes the local roughness of the surface structures, resulting in both chemical and structural heterogeneities. These heterogeneities on molecular scale can be studied using the approach presented in this paper when the Scheutjens–Fleer machinery is more fully implemented. For example, we can easily consider polymer chains pinned at random locations along the surface of the inverse opal. It is even possible to consider polymer brushes on such substrates which may, e.g., preferentially change wetting characteristics of the top surface or the insides of the cavities [47]. Such decorated substrates may feature dramatic hysteresis effects in the contact angle, because the polymers can stabilize the three-phase contact line while the shape of the cavities induces pinning of the contact line. Hence polymers may introduce a second length scale in inverse opal surface structures.

In the current work we have solved the regular solution model using the self-consistent field (SCF) machinery. Only low memory costs were required to find SCF solutions that are linearly proportional to the volume, that is, the number of lattice sites in the system (*M_x_*, *M_y_*, *M_z_*). Also the CPU time scales only linearly with the volume. Here we focused on very small systems and used a desktop PC to find accurate results in a few minutes CPU time. Alternatively, the complete set of equations can be solved on a GPU and using CUDA technology the results may be generated 10 to 100 times faster [48]. As a result systems which are 10 times larger in each direction should still be feasible, while keeping the wall-time for the computations at less than an hour. In this case the regular solution model captures the macroscopically relevant sizes and we do not need to rely on extrapolations.

## Conclusion

Regular solution theory is used to study the wetting behaviour of a simplistic molecular model on a complex inverse opal surface topology. The model features molecular input parameters and gives interfacial energies, contact angles and three-phase contact line shapes. As a result, advancing as well as receding wetting front scenarios were considered. It was found that there is a large contact angle hysteresis in these systems, which was attributed to contact line pinning. We have seen that the cavities can be filled by the liquid or remain dry, i.e., filled by vapour. Cavities in front of the droplet may be filled by a capillary condensation effect, while receding contact angles typically do not empty the liquid filled cavities. Interestingly, when the substrate is slightly hydrophilic it is possible that advancing contact angles have contact angles larger than 90°.

## Supporting Information

File 1 Detailed mathematical calculations.
