# Supplementary material for: Three-gradient regular solution model for simple liquids wetting complex surface topologies
Source: Beilstein J Nanotechnol. 2016 Oct 4;7:1377–96. doi: 10.3762/bjnano.7.129 (PMC5082679; doi:10.3762/bjnano.7.129)
Supplement: File 1 — Detailed mathematical calculations. [file Beilstein_J_Nanotechnol-07-1377-s001.pdf]

Supporting Information

for

Three-gradient regular solution model for simple liquids wetting complex surface topologies

Sabine Akerboom, Marleen Kamperman and Frans A. M. Leermakers*

Address: Physical Chemistry and Soft Matter, Wageningen University, Stippeneng 4, 6708 WE Wageningen, Netherlands

Email: Frans A. M. Leermakers* - frans.leermakers@wur.nl

* Corresponding author

Detailed mathematical calculations

S1 Simple method to solve the regular solution equations efficiently.

Here we consider the SCF route to optimise the regular solution free energy. We take the simplest one-gradient case without surfaces as an example and trust that the reader can generalise the protocol to include surfaces and to account for more than one dimension. The starting point is Equation 6:

$$F = \sum_{-M}^{M} \varphi(z) \ln \varphi(z) + \varphi_{\mathrm{V}}(z) \ln \varphi_{V}(z) + \chi\, \varphi(z)\langle \varphi_{\mathrm{V}}(z)\rangle \qquad \text{(S1)}$$

where we notice that the free energy is a functional of the volume fraction distribution of the liquid and the vapour. We also recall that for each coordinate z we have a compressibility relation

$$\varphi(z) + \varphi_{\mathrm{V}}(z) = 1 \qquad \text{(S2)}$$

In the self-consistent field (SCF) theory the same free energy is expressed in terms of segment densities as well as segment potentials $u(z)$ and $u_{\mathrm{V}}(z)$. We take it that these potentials are dimensionless, that is they are already normalised by the thermal energy $k_{\mathrm{B}}T$. The potentials are going to be used in Boltzmann equations:

$$\begin{aligned} G(z) &= \exp -u(z) \\ G_{\mathrm{V}}(z) &= \exp -u_{\mathrm{V}}(z) \end{aligned} \qquad \text{(S3)}$$

In the self-consistent field theory the starting point of the approach is the mean-field free energy:

$$F = \ln \frac{q^{N}}{N!}\frac{q_{\mathrm{V}}^{N_{\mathrm{V}}}}{N!} - \sum_{z=1}^{M}(u(z)\varphi(z) + u_{\mathrm{V}}(z)\varphi_{\mathrm{V}}(z)) + \sum_{z=1}^{M} \varphi(z)\chi\langle\varphi_{\mathrm{V}}(z)\rangle + \sum_{z=1}^{M} \alpha(z)[\varphi(z) + \varphi_{\mathrm{V}}(z) - 1] \qquad \text{(S4)}$$

It is relatively easy to show that Equations S4 and S1 are the same (we will not prove this here). For this we need to know that the single molecule partition functions q are found by the summation over corresponding Boltzmann weights:

$$\begin{aligned} q &= \sum_{z=1}^{M} G(z) \\ q_{\mathrm{V}} &= \sum_{z=1}^{M} G_{\mathrm{V}}(z) \end{aligned} \qquad \text{(S5)}$$

and the number of liquid and vapour sites is given by

$$\begin{aligned} N &= \sum_{z=1}^{M} \varphi(z) \\ N_{\mathrm{V}} &= \sum_{z=1}^{M} \varphi_{\mathrm{V}}(z) \end{aligned} \qquad \text{(S6)}$$

The optimisation of the free energy (Equation S4) with respect to the segment potentials leads the rule how to compute the volume fractions:

$$\varphi(z) = \frac{N}{q} G(z)$$
$$\varphi_{\mathrm{V}}(z) = \frac{N_{\mathrm{V}}}{q_{\mathrm{V}}} G_{\mathrm{V}}(z) \quad \text{(S7)}$$

Optimisation of the free energy (Equation S4) with respect to the segment density leads to the rule to compute the segment potential:

$$u(z) = \alpha(z) + E(z)$$
$$u_{\mathrm{V}}(z) = \alpha(z) + E_{\mathrm{V}}(z) \quad \text{(S8)}$$

where the interaction contribution to the segment potentials are given by

$$E(z) = \chi\langle\varphi_{\mathrm{V}}(z)\rangle$$
$$E_{\mathrm{V}}(z) = \chi\langle\varphi(z)\rangle \quad \text{(S9)}$$

In summary we thus have the situation that

$$u[\varphi] \underset{\varphi+\varphi_{\mathrm{V}}=1}{\Longleftrightarrow} \varphi[u] \quad \text{(S10)}$$

In words the left hand side says that the segment potentials depend on the volume fractions (that is u given by Equation S8 depends on E which is given in Equation S9 to be a function of φ). The right-hand side of Equation S10 says that the volume fractions in Equation S7 can be computed from the segment potentials, by two routes, namely via the Boltzmann weights (Equation S3) and the partition functions (Equation) S5 which are also traced to Equation S3. We need a numerical scheme to find the so-called SCF solution. We need a fixed point for which the segment potentials both follow from the volume fractions from which they are computed. Or inversely, we need the segment densities which determine the potentials on which they depend. Such a fixed point should obey to the compressibility relation of Equation S2.

Self-consistent field solutions must be generated numerically. Once such a solution is found we can compute the thermodynamic quantities. Of more than average interest there is the grand potential (sum of the surface tensions) Ω_S of the system. It can be shown that $\Omega_S = \sum_{z=1}^{M} \omega(z)$, where the grand potential density is given by

$$\omega(z) = -\alpha(z) - \frac{\chi(\varphi(z)\langle\varphi_{\mathrm{V}}(z)\rangle + \varphi_{\mathrm{V}}(z)\langle\varphi(z)\rangle)}{2} + \chi\varphi^{b}\varphi_{\mathrm{V}}^{b} \quad \text{(S11)}$$

where the bulk volume fractions are found at large values of z or equivalently follow from

$$\varphi^{b} = \frac{N}{q}$$
$$\varphi_{\mathrm{V}}^{b} = \frac{N_{\mathrm{V}}}{q_{\mathrm{V}}} \quad \text{(S12)}$$

S2 Calculation of θ_{kink}

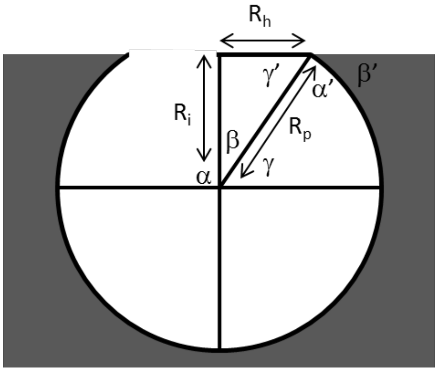

Figure S1: Calculation of θ_{kink} (indicated in the picture as β′).

θ_{kink} can be calculated using $\cos\theta_{kink} = \frac{R_i}{R_p} R_i$ and R_p can be calculated from the input variables for the surface structure (see Table 1): R_i is $(h - R_p)$ and R_p is $\frac{1}{2}d$.

S3 Calculation of Φ_S and *s* at constant Φ_S

To calculate Φ_S, first the area per cavity is calculated for a hexagonal packing.

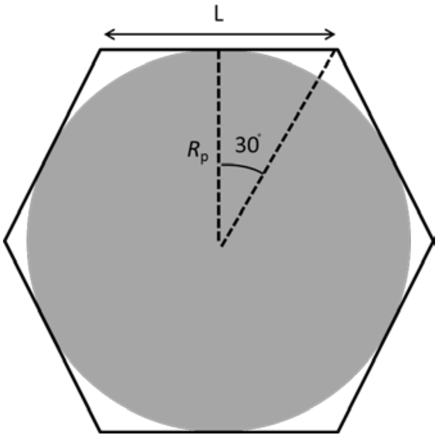

Figure S2: Calculation of area per cavity.

R_p, which is in this case equal to $\frac{1}{2}s$ (Table 1), is related to L via:

$$R_p = \frac{1/2\ L}{\tan(30)} = \frac{\sqrt{3}}{2} L$$

The area per cavity $A_{hexagon}$ is then given by adding the areas of 12 triangles, i.e., 12 times ($1/2 \cdot R_p \cdot 1/2 \cdot L$).

The area of a cavity A_{cavity} is the area of a circle with radius R_h (see S2). Since d and R_i are input parameters, R_h is obtained via Pythagorean theorem, and $A_{cavity} = \pi\ (R_h)^2$

The fraction of solid on the top surface Φ_S is then given by $\Phi_S = 1 - \frac{A_{cavity}}{A_{hexagon}}$.

To calculate s for different c at constant Φ_S, first, a constant Φ_S has to be defined. This sets the ratio A_{cavity}:$A_{hexagon}$. The cavity size depends on the value of c, and since the ratio is fixed, $A_{hexagon}$ is obtained per c. This area is used to calculated to an effective $R_{p,effective}$, and next a surface can be created with $s = 2 \cdot R_{p,effective}$.
